# A Matched Case-Control Study of Noncholesterol Sterols and Fatty Acids in Chronic Hemodialysis Patients

**DOI:** 10.3390/metabo11110774

**Published:** 2021-11-12

**Authors:** Marek Vecka, Magdalena Dušejovská, Barbora Staňková, Ivan Rychlík, Aleš Žák

**Affiliations:** 1 Fourth Department of Internal Medicine, First Faculty of Medicine, Charles University and General University Hospital in Prague, U Nemocnice 2, 128 08 Prague, Czech Republic; M.Dusejovska@seznam.cz (M.D.); barsta@atlas.cz (B.S.); zak.ales@email.cz (A.Ž.); 2 Institute of Clinical Chemistry and Laboratory Diagnostics, First Faculty of Medicine, Charles University and General University Hospital in Prague, Na Bojišti 3, 121 08 Prague, Czech Republic; 3 Department of Internal Medicine, Third Faculty of Medicine, Charles University and University Hospital Královské Vinohrady, Šrobárova 50, 100 34 Prague, Czech Republic; ivan.rychlik@gmail.com

**Keywords:** hemodialysis, non-cholesterol sterols, fatty acids, chronic kidney disease, hypolipidemic treatment

## Abstract

Dyslipidemia is common among patients on hemodialysis, but its etiology is not fully understood. Although changes in cholesterol homeostasis and fatty acid metabolism play an important role during dialysis, the interaction of these metabolic pathways has yet to be studied in sufficient detail. In this study, we enrolled 26 patients on maintenance hemodialysis treatment (high-volume hemodiafiltration, HV HDF) without statin therapy (17 men/9 women) and an age/gender-matched group of 26 individuals without signs of nephropathy. The HV-HDF group exhibited more frequent signs of cardiovascular disease, disturbed saccharide metabolism, and altered lipoprotein profiles, manifesting in lower HDL-C, and raised concentrations of IDL-C and apoB-48 (all *p* < 0.01). HV-HDF patients had higher levels of campesterol (*p* < 0.01) and β-sitosterol (*p* = 0.06), both surrogate markers of cholesterol absorption and unchanged lathosterol concentrations. Fatty acid (FA) profiles were changed mostly in cholesteryl esters, with a higher content of saturated and n-3 polyunsaturated fatty acids (PUFA) in the HV-HDF group. However, n-6 PUFA in cholesteryl esters were less abundant (*p* < 0.001) in the HV-HDF group. Hemodialysis during end-stage kidney disease induces changes associated with higher absorption of cholesterol and disturbed lipoprotein metabolism. Changes in fatty acid metabolism reflect the combined effect of renal insufficiency and its comorbidities, mostly insulin resistance.

## 1. Introduction

Lowering LDL cholesterol concentration is widely accepted as an effective tool for reducing cardiovascular mortality. However, statin treatment is not completely successful in mitigating cardiovascular complications among patients on maintenance dialysis therapy. Dialyzed patients often suffer from dyslipidemia characterized by elevated levels of very-low-density lipoprotein (VLDL) and intermediate-density lipoprotein (IDL) cholesterol, low HDL cholesterol, and high concentrations of triacylglycerols (TAG) [1].

However, there is a scarcity of data on the long-term effects of dialysis on lipid concentrations, while rare studies of cholesterol homeostasis [2,3,4] have produced discrepant results. In the literature, concentrations of lathosterol (a surrogate marker of cholesterol biosynthesis) are reported to be lowered or unchanged, with concentrations of phytosterols (a surrogate marker of cholesterol absorption) usually elevated or unchanged. Moreover, phytosterols interfere with cholesterol absorption in the gut, thus being able to lower plasma cholesterol. This effect is useful for the reduction of LDL cholesterol levels in dyslipidemic patients as a part of nonpharmacological therapy and also as a supplement to statin/ezetimibe treatment.

Dialyzed patients with increased cholesterol absorption are at higher mortality risk [3], which may explain the low efficacy of statin treatment in end-stage kidney disease (ESKD) as documented in the AURORA and 4D studies [5,6]. Nonetheless, one study found that in cases where cholesterol absorption was limited by combined ezetimibe/statin therapy, the risk of atherosclerotic events decreased in patients with chronic kidney disease (CKD) but not in an ESKD subgroup [7]. Renal tissue can amass cholesterol via receptors [8] or biosynthesis [9]. Disturbed cholesterol homeostasis can also have a detrimental effect on the kidneys, as cholesterol overload in the kidneys contributes to the suppression of cell proliferation in the renal tubules, causing chronic kidney damage [10]. Chronic kidney damage is also connected with reduced fatty acid (FA) oxidation, including fibrotic responses during tubulo-interstitial damage [11]. Both FA and cholesterol have been linked to the progression of renal disease via lipotoxicity [12] or regulation of kidney fibrosis connected with microRNA-33 [13]. In addition, FA metabolism is associated with cholesterol homeostasis via several other mechanisms, including the turnover of lipoproteins in the circulation, where the liver plays the most important role. Systemic cholesterol delivered through LDL particles is regulated in the liver in two different ways: (1) via changes in the secretion and biosynthesis of bile acids, which consequently modulate cholesterol absorption in the intestinal lumen (hence, cholesterol liver input) and (2) via redistribution of cholesterol between free and esterified pools, dictated by the type of long-chain FA that reach hepatic tissue [14].

The prominent role of HDL lipoproteins in reverse cholesterol transport is partly linked to the formation of fatty acid esters of cholesterol molecules during the remodeling of nascent HDL particles. This process is impaired in patients with nephrotic syndrome and/or ESKD [15]. Cholesterol in HDL lipoproteins can also equilibrate with erythrocytes [16]. In hemodialyzed patients, these blood elements, which exhibit higher turnover and cell distribution width, are associated with increased mortality [17] and higher accessibility of the cholesterol pool [16]. Very recently, both cholesterol and fatty acids were linked to the severity of depression in chronic kidney disease [18].

The possible interaction between cholesterol and fatty acid metabolism can be assessed in several ways, including accounting for the FA profiles of plasma cholesteryl esters. To our knowledge, a concomitant analysis of FA metabolism and cholesterol homeostasis has not yet been published. Therefore, the aim of our study was to depict differences in fatty acid and cholesterol metabolism between hemodialyzed patients and individuals with proper renal function. Our hypothesis is that the metabolism of sterols and fatty acids is interconnected and susceptible to alteration through disturbed lipoprotein turnover in hemodialyzed patients.

## 2. Results

### 2.1. Biochemical and Anthropometric Parameters

There were no differences between the HV-HDF group and non-dialysed individuals without signs of CKD (control group) with regard to the conventional risk factors for atherosclerotic cardiovascular disease (ASCVD) (age, sex ratio, presence of hypertension, DM (diabetes mellitus), dyslipidaemia). However, the HV-HDF group exhibited more frequent signs of clinically manifested ASCVD (54% vs. 8%; *p* = 0.001). Concentrations of total cholesterol were lower in the HV-HDF group, mainly due to a decreased content of HDL cholesterol. Both groups had comparable concentrations of apolipoprotein A-I and apo B, but the HV-HDF group had almost six-fold higher concentrations of apoB-48. The parameters of saccharide metabolism in the HV-HDF group were characterised by higher levels of glucose, insulin, and the HOMA-IR index (see Table 1).

### 2.2. Changes in Plasma Non-Cholesterol Sterols

Dialysed patients had higher levels of the phytosterol campesterol and borderline high β-sitosterol, both surrogate markers of cholesterol absorption (Table 2) related to total cholesterol concentration. Differences in lathosterol concentrations did not reach statistical significance.

### 2.3. Fatty Acid Profiles of the Major Plasma Lipid Classes

The following three major plasma lipid classes were analyzed for fatty acid content: phospholipids (PL), triacylglycerols (TAG), and cholesteryl esters (CE) (Table 3). In the HV-HDF group only, the content of saturated FA (SFA) was higher in CE due to the higher content of palmitic acid (16:0). The content of another major saturated fatty acid, stearic acid (18:0), was lower in the HV-HDF group. In this group, the ratio of monounsaturated fatty acids (MFA) was lower in plasma TAG only. The content of the major MFA, oleic acid (18:1n-9), was lower in both TAG and CE. Minor MFA, including vaccenic acid (18:1n-7), palmitoleic acid (16:1n-7), and hypogeic acid (16:1n-9), decreased inconsistently across the plasma lipid classes analysed.

Polyunsaturated fatty acids (PUFA) were less abundant in lipid classes such as n-6 PUFA in the HV-HDF group, with a tendency to increase the content of n-3 PUFA. These changes were caused by a reduced content of linoleic (18:2n-6) and increased content of eicosapentaenoic (20:5n-3) and docosahexaenoic (22:6n-3) acids. The content of arachidonic acid (20:4n-6) increased in the HV-HDF group. The prevalence of the essential FA deficiency index (defined as the ratio 16:1n-9/18:2n-6 ≥ 0.086) was observed for plasma TAG only in one individual in both groups. For PL and CE, no individuals met the above criteria (data not presented).

### 2.4. Correlations between Sterols and Fatty Acids

Spearman’s rank-order correlations between non-cholesterol sterol and molar percentages of FA in individual lipid classes are presented in Supplementary Tables S1–S3. In the pooled groups, we observed positive correlations of campesterol/TC and β-sitosterol/TC with myristic (14:0) and docosapentaenoic (22:5n-3) acids in all lipid classes and with palmitoleic acid (18:1n-7) in plasma CE as well as in PL. These positive correlations reached statistical significance only in TAG for AA (20:4n-6) and in CE for DHA (22:6n-3). In CE, we also observed a negative correlation for LA (18:2n-6).

The lathosterol/TC ratio correlated positively with ALA (18:3n-3) in all lipid classes. In PL, there were positive correlations with myristic (14:0), palmitic (16:0), and palmitoleic (16:1n-9) acids, but the content of arachidonic (20:4n-6) and stearic (18:0) acids correlated negatively with the lathosterol/TC ratio.

## 3. Discussion

Patients on chronic hemodialysis treatment displayed lower total cholesterol, caused by lower concentrations of HDL-C and VLDL-C and higher concentrations of TAG. In both study groups, patients exhibited dyslipidemia (only one patient in the HV-HDF group had no dyslipidemia). Cholesterol is usually redistributed from VLDL to IDL and LDL particles in hemodialyzed patients [19]. This study tried to describe cholesterol and fatty acid metabolism in more detail in individuals without statin therapy. Compared to our previous study [19], we observed a less pronounced redistribution effect, with only IDL particles exhibiting a higher cholesterol content in the HV-HDF group. Interestingly, most changes in fatty acid profiles were observed in cholesteryl esters, the lipid class formed in plasma during the metabolism of lipoprotein particles that is known to be impaired in hemodialyzed patients [15].

Typically, hemodialysis patients without statin/ezetimibe treatment do not have high concentrations of cholesterol. LDL-C concentrations are usually low in ESKD patients [5,6,7], while decreased LDL-C concentrations are influenced by body weight reduction [20], malnutrition, and systemic inflammation [21]. Another factor lowering LDL-C concentration could be the use of phosphate binders that are known to change lipoprotein profiles [22], but we did not observe the effect of binder use on anthropometric or lipid parameters with the exception of lower concentrations of apoB in the binder group (data not shown). Our group of chronic dialysis patients exhibited increased concentrations of hs-CRP and SAA, which are inflammatory markers as well as important predictors of mortality in chronic dialysis patients [23]. In this study, the higher content of cholesterol in LDL particles was caused by the shift of cholesterol into IDL particles that are usually included in assays of LDL cholesterol. Furthermore, higher levels of LDL-C are associated with long-term survival in hemodialysis patients [24], and our group of dialyzed patients was maintained on hemodialysis for 4 years on average, which is an indicator of successful treatment and favorable metabolic status in patients of this age (see also Supplementary Tables S4 and S5). This effect probably prevailed over the LDL-C lowering effect of increased inflammatory stress.

In our study, concentrations of surrogate markers for cholesterol absorption were higher in the HV-HDF group, which may have been caused by several different mechanisms, e.g., either a simple response to lowered cholesterol biosynthesis [25] or overall differences in biosynthesis/absorption equilibrium. Blood samples were drawn from all patients at a similar time during the day, as cholesterol biosynthesis precursors and C4 concentrations (unlike phytosterols) are influenced by diurnal rhythm [26,27], as demonstrated even in one model of experimentally-induced renal failure [28]. Nevertheless, lathosterol concentrations were similar in both groups, indicating no change in cholesterol biosynthesis. It is also possible that another factor, such as the accumulation and mobilization of phytosterols in adipose tissue, influenced the interplay between biosynthesis and absorption [29,30]. Adipose tissue in CKD patients not only contains a large number of small adipocytes but also exhibits characteristic features of inflammatory phenotypes [31]. Bile acid biosynthesis is another metabolic process that influences cholesterol turnover; however, we found no associations between phytosterol concentrations and C4, a surrogate marker of bile acid biosynthesis (data not presented). In the dialysis group, the lathosterol/TC ratio positively correlated with C4 concentration (r = 0.805; *p* < 0.001, Spearman’s rank-order correlation coefficient). However, the literature contains conflicting results: one study reported high cholesterol biosynthesis [32] using mevalonate as a marker of biosynthesis, while another, which excluded phytosterols from its analysis, found lower lathosterol in hemodialyzed patients [33].

Higher concentrations of apoB-48 in the HV-HDF group prove higher numbers of chylomicron remnants, implicating impaired clearance and/or increased intestinal production of phytosterol-containing chylomicrons [34]. Higher indices of cholesterol absorption were accompanied by a higher incidence of ASCVD in the HV-HDF group. In several studies, the campesterol/lathosterol ratio has been related to the progression of coronary atherosclerosis expressed as coronary plaque vulnerability [35,36].

It is also possible that increased cholesterol absorption markers are caused by upregulation of the Niemann-Pick C1-like 1 protein (NPC1L1), which transports sterols from the intestinal lumen to enterocytes. Production of this protein is suppressed by peroxisome proliferator-activated receptors (PPARα), which is downregulated during uremia [37] in ESKD patients and during insulin-resistant states [38,39]. Consequently, our group of chronic dialysis patients exhibited increased levels of urea and the HOMA insulin resistance index. However, at higher HOMA index levels, surrogate marker values of cholesterol homeostasis have been shown to be independent of IR levels (Vecka et al., unpublished data).

Concomitant changes in sterol and FA plasma profiles can reflect impaired esterification processes in various tissues. Within enterocytes, esterification of FA into CE by acyl-CoA cholesterol acyltransferase isoform 2 (ACAT-2) is supported by processes that promote the retention of cholesterol, such as the higher activity of the NPC1L1 transporter and/or ACAT-2 as well as lower activity of ATP-binding cassette transporters ABCG5/G8, which are co-transporters of sterols from enterocytes to the intestinal lumen. In rats, the removal of one kidney changed cholesterol homeostasis in the remaining kidney, leading to reduced biosynthesis and increased esterification by ACAT-1 [40].

The distribution of FA in dialysis patients is characterized by a higher saturated FA (SFA) content and a lower ratio of polyunsaturated FA (PUFA) [41], hallmarks of a diabetic FA profile [42] with deficiency of essential FA. CKD progression is linked with plasma PUFA levels [43], while the faster CKD progression relates to low PUFA concentration [44]. Although fatty acid profiles in ESKD patients are often compared to healthy populations, comparisons with specific pathologic states, such as DM, are uncommon [45]. In our study, most changes in FA profiles were observed for plasma CE. One study identified diacylglycerols and CE as lipid classes with the highest predictive power for the progression of CKD to ESKD [46].

In the HV-HDF group, the content of EPA and DHA was higher in CE and TAG in agreement with previous studies [41,45]. Lower content of n-6 PUFA was caused by lower levels of linoleic acid (LA, 18:2n-6), a feature that has also been observed in diabetic patients when compared with a dialysis patient group [45]. Although healthy individuals have been shown to exhibit lower concentrations of arachidonic acid (AA, 20:4n-6) [41], the lower LA content we observed may have stemmed from higher levels of oxidative stress, higher metabolization of LA to AA/prostanoids, or decreased dietary intake. Both CKD and ESKD are states with an increased level of oxidative stress. Compared to LA/AA (members of the n-6 PUFA family), n-3 PUFAs have a higher ability to resist oxidative modifications in lipid structures exposed to the polar environment. The n-3 PUFA chains form conformations protective against the propagation of oxidative changes [47], which was confirmed by the effect of dietary supplementation on the oxidation of LDL particles [48]. The higher content of PUFAn-3 observed in the HV-HDF group can result from this phenomenon.

Despite the identical prevalence of DM in both groups, we observed more pronounced parameters of disturbed saccharide metabolism in the HV-HDF group, such as higher values of the HOMA index, which reflects impaired insulin resistance. Higher SFA content is typical for both insulin-resistant [49] and ESKD patients [50], a trend also observed in our study. Palmitic acid (16:0) inhibits glucose clearance in podocytes and insulin-sensitive cells [50]. Conversely, the content of another saturated fatty acid, stearic acid, was lower across all plasma lipid classes in our HV-HDF patients.

In dialysis patients, monounsaturated fatty acids are more abundant in erythrocyte membranes [51], while MFA content is more influenced by insulin resistance [49] than ESKD [45]. In our HV-HFD group, we observed lower MFA content only in TAG due to decreased content of oleic acid (18:1n-9). The activity of delta 9 desaturase (D9D), estimated as the 18:1n-9/18:0 ratio, was higher in the HV-HDF group across all lipid classes, probably due to the positive influence of insulin resistance on D9D [49]. Insulin-resistant states such as obesity, type 2 DM, metabolic syndrome, and chronic pancreatitis are characterized by higher activity of D6D and lower activity of D5D [51,52,53,54]. In patients with ESKD, we found higher indices of D5D activity only, as previously demonstrated in pancreatic cancer patients [55].

This study is limited by the relatively low number of individuals enrolled and the absence of data on the dietary intake of sterols and fatty acids, bile acid concentrations, and the activities of some proteins linked to the metabolism of steryl esters in circulation (e.g., lecitin-cholesterol acyltransferase). The strengths of the study are the analysis of several lipid classes containing sterols and fatty acids and the matched design aimed at minimizing confounding factors.

## 4. Materials and Methods

### 4.1. Patients

We recruited patients treated by chronic high-volume hemodiafiltration therapy (HV-HDF) at a dialysis unit between the years 2013 and 2017. From a total of 42 individuals on HV-HDF treatment meeting the eligibility criteria, 10 were excluded based on age (>85 years or <20 years) and 6 due to incomplete or unavailable biochemical data. In all, 26 patients treated by HV-HDF (17M/9F, aged 63.0 ± 12.9; mean ± SD, years) were included for further study. The median vintage of all patients on dialysis therapy was 3.9 years (interquartile range: 2.2–6.8 years). At baseline, all patients exhibited stable, 3 had anuria, and 8 had type 2 diabetes mellitus (plus insulin analogue therapy in 5 cases). As a phosphate binder, Renagel^®^ (sevelamer hydrochloride, Genzyme Europe BV, Amsterdam, The Netherlands) was used in 16 patients. For all patients, the dialysis treatment time was 12–15 h/week. Dialysis efficacy, expressed by online clearance monitoring (OCM), reached values of 1.58 ± 0.25 (mean ± SD). The median interdialysis weight gain was 2.8 kg.

Exclusion criteria were as follows: serious endocrinopathy, pregnancy and breastfeeding, alcohol abuse, and acute inflammatory disease. We also excluded individuals on statin therapy, which is known to affect concentrations of non-cholesterol sterols [56], and those treated with other lipid-lowering drugs (fibrates, niacin) or taking PUFA n-3 supplements.

The age- and sex-matched control group (individuals without signs of nephropathy, CON) consisted of 26 probands (17M/9F, age 61.3 ± 8.6 years). None of these patients had signs of chronic kidney disease (CKD) (eGFR was calculated according to the CKD-EPI formula [57]). All individuals remained in a stable (>1 year) metabolic state with no changes in therapy. The study was approved by the ethics committee of the participating institution and conducted according to the Declaration of Helsinki principles. All participants received detailed information on the research involved and provided their informed signed consent. The basic characteristics of both groups are presented in Table 1.

### 4.2. Methods

Blood samples were drawn after overnight fasting; in the HV-HDF group, samples were obtained before the dialysis procedure (after a 2-day delay between the HV-HDF procedures, including 8–12 h of fasting). Basic biochemical parameters were analyzed using standard colorimetric/enzymatic methods. All dialyzed patients underwent HV-HDF treatment on the Fresenius 5008 CorDiax system (Fresenius Medical Care, Bad Homburg, Germany). Cholesterol content in subfractions of plasma LDL and HDL was analysed using high-performance discontinuous gel electrophoresis with polyacrylamide tube gel (Lipoprint^®^ LDL/HDL System, Quantimetrix, Redondo Beach, CA, USA) [19]. Concentrations of serum amyloid A (SAA), apoB-48, and high-sensitivity C-reactive protein (hs-CRP) were obtained using commercial ELISA kits. Total plasma concentrations of non-cholesterol sterols were assayed after direct saponification of 250 μL plasma with 5 mL of 1 M methanolic KOH, extraction with 2 × 2 mL of hexane and evaporated residue was subjected to derivatization into carbamates using epicoprostanol as the internal standard. Concentrations of non-cholesterol sterols were analysed on the LC-MS/MS platform using a 150 × 2.1 mm, 3 μm HYPERSil GOLD column (Thermo Scientific, Waltham, MA, USA) [58].

Fatty acids were analysed in all major plasma lipid classes (cholesteryl esters, triacylglycerols, and phospholipids) after modified Folch extraction of 1000 μL plasma. Separation was performed by TLC and direct transmethylation with 1 M methanolic sodium methoxide at an ambient temperature [59] or at 80 °C for cholesteryl esters. FA profiles of plasma lipid classes were assayed using gas chromatography on the same device used for sterols. The column used was the SLB^TM^-IL111i (60 m × 0.25 mm ID, 0.20-μm df) (Supelco, Bellefonte, PA, USA) in conjunction with a temperature program starting at 50 °C with a 1-min hold, then raised to 130 °C at 30 K/min, followed by gradients of 20 K/min to 160 °C, 10 K/min to 170 °C with a 10-min hold, 1 K/min to 180 °C with an 8-min hold and 2 K/min to 220 °C. Injector and detector temperatures were set at 260 °C in split-injection mode (1:20). Retention times for all sterols and FA methyl esters analysed were verified using commercially available standards (Sigma Aldrich, NuChek Prep Inc., Elysian, MN, USA). Concentrations of 7α-hydroxy-4-cholesten-3-one (C4) were analysed on the LC-MS/MS platform using a 150 × 2.1 mm, 3 μm HYPERSil GOLD column (Thermo Scientific, Waltham, MA, USA) [60].

STATISTICA CZ ver.12 (StatSoft Inc., Tulsa, OK, USA) software was used for the statistical processing of results. Non-normally distributed variables were log-transformed. The significance of differences between groups was assessed by ANCOVA, with age and BMI as covariates. Distribution differences were calculated using the chi-square test with Yates’ correction. Values of *p* < 0.05 were considered statistically significant.

## 5. Conclusions

To conclude, we prove in this study that hemodialysis treatment of end-stage renal disease induces changes in the metabolism of cholesterol and phytosterols, which relate to higher absorption of cholesterol and disturbed lipoprotein metabolism. The changes we observed in fatty acid metabolism highlight the combined effect of renal insufficiency and the presence of comorbidities such as insulin resistance. These findings may contribute to delivering more effective DLP treatment aimed at increasing cholesterol absorption (ezetimibe) in chronic dialysis patients.

## Figures and Tables

**Table 1:** Basic anthropometric and selected biochemical parameters of the studied groups.

	Group	
Parameter	CON (*n* = 26)	HV-HFD (*n* = 26)
Age (years)	61.3 ± 8.6	63.0 ± 12.9 ^1^
Dialysis vintage (years)	NA	3.9 [2.2–6.8]
Body weight (kg) *	87.7 ± 20.6	76.3 ± 22.2
BMI (kg/m^2^)	29.3 ± 5.3	24.7 ± 6.1
Males/females	17/9	17/9
Diabetes mellitus [*n* (%)]	8(31)	8(31)
Hypertension [*n* (%)]	21(81)	23(88)
Smoking [*n* (%)]	2(8)	7(27)
Cardiovascular disease [*n* (%)]	2(8)	14(54) ^b^ ***
eGFR (ml/s)	1.41 ± 0.24	0.13 ± 0.04 ^a^ ***
Total cholesterol (mmol/L)	5.64 ± 1.26	4.95 ± 1.12 *
HDL-C (mmol/L)	1.48 ± 0.50	1.20 ± 0.34 ***
nonHDL-C (mmol/L)	4.17 ± 1.05	3.75 ± 1.10
VLDL (%C)	24.6 ± 7.8	19.4 ± 4.6
Total LDL (%C)	54.2 ± 7.7	60.0 ± 5.2 *
IDL (IDL_A-C_) (%C)	26.3 ± 6.6	31.0 ± 4.6 *
Large LDL (LDL_1–2_) (%C)	25.0 ± 7.1	26.2 ± 6.7
Small LDL (LDL_3–7_) (%C)	1.2 [0.7–4.3]	1.8 [1.1–3.3]
Sum of HDL fractions (%C)	20.9 ± 5.9	18.3 ± 6.0 *
Large HDL (HDL_1–3_) (%C in HDL)	22.3 ± 6.9	30.5 ± 11.8
Intermediate HDL (HDL_4–7_) (%C in HDL)	43.8 ± 3.4	46.2 ± 5.3 *
Small HDL (HDL_8–10_) (%C in HDL)	33.9 ± 8.1	23.3 ± 10.9 **
Non-esterified fatty acids (mmol/L)	0.49 [0.37–0.67]	0.31 [0.15–0.64] *
Triacylglycerols (mmol/L)	1.20 [0.93–1.57]	1.88 [1.03–2.53] **
Glucose (mmol/L)	5.30 [4.90–5.60]	6.12 [5.10–7.05] **
Insulin (mU/L)	9.9 [6.1–16.9]	14.4 [9.0–34.0] **
HOMA-IR index	2.4 [1.4–4.0]	4.0 [2.0–10.2] **
Apo A-I (g/L)	1.50 ± 0.41	1.43 ± 0.94
Apo B (g/L)	1.21 ± 0.28	1.06 ± 0.40
Apo B-48 (mg/L)	7 [4–16]	40 [30–54] ***
Lp(a) (nmol/L)	38 [10–68]	48 [24–103]
SAA (mg/mL)	22 [14–32]	40 [15–114] *
C4 (ng/mL)	17 [15–32]	25 [14–48]
Uric acid (μmol/L)	342 ± 77	330 ± 74
Urea (mmol/L)	5.3 ± 0.8	16.5 ± 5.5 ***
hs-CRP (mg/L)	3.4 [1.2–5.0]	9.5 [5.0–13.4] ***

^1^ Data are given in average ± SD or median [Q1–Q3] format; ^a^ ANCOVA adjusted for age and BMI; ^b^ χ^2^ test with Yates’ correction: *—*p* < 0.05, **—*p* < 0.01, ***—*p* < 0.001; DM—Diabetes mellitus, HDL—High-density lipoprotein, hs-CRP—High-sensitivity C-reactive protein, IDL—Intermediate-density lipoprotein, LDL—Low-density lipoprotein, SAA—Serum amyloid A, TC—Total cholesterol, VLDL—Very-low-density lipoprotein; HOMA-IR index = insulin (mU/L)*glucose (mmol/L)/22.5, %C—% of cholesterol.

**Table 2:** Concentrations of non-cholesterol sterols in plasma.

	Group	
Non-Cholesterol Sterol	CON (*n* = 26)	HV-HFD (*n* = 26)
Lathosterol (μmol/L)	8.84 ± 5.33	8.83 ± 4.56
Campesterol (μmol/L)	9.01 ± 3.42	11.06 ± 5.16 *
β-sitosterol (μmol/L)	6.83 ± 3.11	7.34 ± 3.21
Lathosterol/TC (μmol/mmol)	1.55 ± 0.71	1.90 ± 1.10
Campesterol/TC (μmol/mmol)	1.68 ± 0.75	2.26 ± 0.95 **
β-sitosterol/TC (μmol/mmol)	1.26 ± 0.61	1.53 ± 0.63 +

Data are given in average ± SD; ANCOVA adjusted for age and BMI: + *p* = 0.06, * *p* < 0.05, ** *p* < 0.01; TC—Total cholesterol.

**Table 3:** Fatty acid profiles in plasma lipid classes.

Fatty Acid	Phospholipids (PL)		Triacylglycerols (TAG)		Cholesteryl Esters (CE)	
	CON	HV-HFD	CON	HV-HFD	CON	HV-HFD
14:0 *	**0.24 ± 0.10**	**0.30 ± 0.07 +**	**1.24 ± 0.46**	**2.67 ± 0.81 +++**	**0.34 ± 0.25**	**0.95 ± 0.25 +++**
16:0	32.50 ± 4.57	33.85 ± 2.20	27.19 ± 3.87	27.50 ± 2.57	**10.36 ± 1.86**	**14.29 ± 1.34 +++**
16:1n-9	0.14 ± 0.03	0.14 ± 0.03	0.86 ± 0.23	0.79 ± 0.15	**0.44 ± 0.15**	**0.57 ± 0.13 ++**
16:1n-7	0.60 ± 0.25	0.53 ± 0.20	3.33 ± 0.86	3.37 ± 1.03	2.66 ± 1.05	3.05 ± 1.10
18:0	**14.42 ± 1.67**	**13.11 ± 1.09 ++**	**4.00 ± 0.84**	**3.12 ± 0.56 +++**	1.01 ± 0.31	0.86 ± 0.35
18:1n-9	10.94 ± 1.26	10.77 ± 1.42	**40.44 ± 2.77**	**38.47 ± 4.04 +**	**21.27 ± 1.90**	**19.85 ± 1.98 ++**
18:1n-7	**1.53 ± 0.13**	**1.78 ± 0.31 ++**	2.50 ± 0.35	2.34 ± 0.42	1.29 ± 0.15	1.41 ± 0.26
18:2n-6	**22.92 ± 2.05**	**20.35 ± 2.85 ++**	15.96 ± 3.01	16.00 ± 3.75	**55.23 ± 3.59**	**48.22 ± 4.41 +++**
18:3n-6	0.10 ± 0.05	0.07 ± 0.03	0.28 ± 0.11	0.32 ± 0.15	0.82 ± 0.32	0.77 ± 0.32
18:3n-3	0.25 ± 0.12	0.23 ± 0.07	**0.94 ± 0.33**	**1.44 ± 0.45 +++**	0.71 ± 0.22	0.74 ± 0.14
20:2n-6	**0.41 ± 0.13**	**0.50 ± 0.11 +**	**0.31 ± 0.09**	**0.16 ± 0.04 +++**	0.09 ± 0.04	0.11 ± 0.05
20:3n-6	2.70 ± 0.84	2.56 ± 0.53	**0.34 ± 0.17**	**0.24 ± 0.07 +**	0.71 ± 0.20	0.63 ± 0.11
20:4n-6	**8.77 ± 2.73**	**10.28 ± 1.54 +**	1.30 ± 0.52	1.31 ± 0.31	**4.46 ± 1.69**	**6.40 ± 1.33 +++**
20:5n-3	1.13 ± 0.62	1.23 ± 0.90	**0.20 ± 0.11**	**0.30 ± 0.33 +**	**0.31 ± 0.23**	**0.79 ± 0.39 +++**
22:5n-6	**0.12 ± 0.06**	**0.17 ± 0.05 ++**	**0.06 ± 0.03**	**0.08 ± 0.03 +**	**0.02 ± 0.01**	**0.03 ± 0.02 ++**
22:5n-3	**0.58 ± 0.34**	**0.92 ± 0.27 +++**	**0.17 ± 0.10**	**0.33 ± 0.16 +++**	**0.02 ± 0.02**	**0.07 ± 0.04 +++**
22:6n-3	2.24 ± 1.41	2.70 ± 1.04	0.38 ± 0.25	0.46 ± 0.51	**0.08 ± 0.06**	**0.32 ± 0.12 +++**
ΣSFA	47.24 ± 3.91	47.32 ± 1.62	32.53 ± 4.41	33.82 ± 2.96	**11.76 ± 2.12**	**16.20 ± 1.53 +++**
ΣMFA	13.36 ± 1.39	13.40 ± 1.79	**47.45 ± 2.71**	**45.42 ± 4.20 +**	25.77 ± 2.58	25.68 ± 3.30
ΣPUFAn-6	35.21 ± 3.23	34.21 ± 2.27	18.34 ± 3.19	18.25 ± 3.61	**61.35 ± 4.17**	**56.21 ± 4.07+++**
ΣPUFAn-3	4.20 ± 2.14	5.07 ± 1.95	**1.69 ± 0.45**	**2.52 ± 1.21 +++**	**1.12 ± 0.29**	**1.91 ± 0.69 +++**
D9D16	0.02 ± 0.01	0.02 ± 0.01	0.12 ± 0. 03	0.12 ± 0.04	**0.25 ± 0.08**	**0.21 ± 0.08 +**
D9D18	0.77 ± 0.13	0.83 ± 0.14	**10.5 ± 2.2**	**12.7 ± 2.5 ++**	22.9 ± 6.6	25.8 ± 8.3
D6D	0.004 ± 0.002	0.004 ± 0.002	0.018 ± 0.008	0.021 ± 0.011	0.015 ± 0.006	0.016 ± 0.008
D5D	**3.36 ± 0.84**	**4.22 ± 1.27 +**	**3.92 ± 0.62**	**5.69 ± 1.54 +++**	**6.3 ± 2.0**	**10.6 ± 3.3 +++**

Data (molar percentages) are given in average ± SD format. Symbols and abbreviations: * shorthand notation for fatty acids: carbon number: double bond number, n—Position of carbon with first double bond from methyl end; FA—Fatty acids, ∑—Sum, SFA—Saturated fatty acids, MFA—Monounsaturated fatty acids, n-6 PUFA—Polyunsaturated fatty acids of the n-6 family, n-3 PUFA—Polyunsaturated fatty acids of the n-3 family, D9D16—D9 desaturase for C16 (16:1n-7/16:0), D9D18—D9 desaturase for C18 (18:1n-9/18:0), D6D—D6 desaturase (18:3n-6/18:2n-6), D5D—D5 desaturase (20:4n-6/20:3n-6); significant difference (ANCOVA adjusted for age and BMI) from the non-HV-HDF group: + *p* < 0.05; ++ *p* < 0.01; +++ *p* < 0.001. The differences are highlighted in bold.

## Data Availability

All the data supporting the findings of this study are included in this article and the Supplementary Materials.

## Supplementary Materials

The following are available online at https://www.mdpi.com/article/10.3390/metabo11110774/s1, Table S1: Correlations between fatty acid content and noncholesterol sterols in plasma phospholipids, Table S2: Correlations between fatty acid content and noncholesterol sterols in plasma triacylglycerols, Table S3: Correlations between fatty acid content and noncholesterol sterols in plasma cholesteryl esters, Table S4: Selected parameters of liver function, Table S5: Relationships between nutritional parameters and indices of inflammation.
