# Supplementary material for: A Matched Case-Control Study of Noncholesterol Sterols and Fatty Acids in Chronic Hemodialysis Patients
Source: Metabolites. 2021 Nov 12;11(11):774. doi: 10.3390/metabo11110774 (PMC8618803; doi:10.3390/metabo11110774)
Supplement: Supplementary file 1 [file metabolites-11-00774-s001.zip › metabolites-1455898-supplementary.pdf]

Supplementary Table S1

Correlations between fatty acid content and noncholesterol sterols in plasma phospholipids

fatty acid	lathosterol/TC (mmol/mmol)	campesterol/TC (mmol/mmol)	b-sitosterol/TC (mmol/mmol)
14:0*	0.2186+	0.1527+	0.1176
16:0	0.2137+	-0.0025	-0.0491
16:1n-9	0.1690+	0.0103	0.0081
16:1n-7	-0.0156	-0.0895	-0.0982
18:0	-0.1567+	-0.0736	-0.0321
18:1n-9	-0.0588	0.0042	0.0277
18:1n-7	0.0025	0.1887+	0.1672+
18:2n-6	0.0025	-0.0964	-0.0297
18:3n-6	-0.0956	-0.2622+	-0.2514+
18:3n-3	0.1889+	0.0758	0.0176
20:2n-6	-0.1177	0.0875	0.1291
20:3n-6	-0.0798	-0.0643	-0.0444
20:4n-6	-0.1875	0.0883	0.0604
20:5n-3	0.0462	0.0466	-0.0279
22:5n-6	-0.1023	0.1257	0.1189
22:5n-3	0.0367	0.2206+	0.1528+
22:6n-3	-0.0013	0.1139	0.0815
SSFA	0.1852+	-0.0704	-0.1072
SMUFA	-0.0616	0.0233	0.0395
SPUFAn-6	-0.1584+	0.0022	0.0536
SPUFAn-3	0.0209	0.1397	0.0856
D9D16	-0.0902	-0.1005	-0.0840
D9D18	0.0646	0.0524	0.0426
20:5n-3/20:4n-6	0.1395	-0.0115	-0.0721
22:6n-3/20:4n-6	0.1116	0.0225	-0.0024

*Data for fatty acids are given in molar percentages. Symbols and abbreviations: * -shorthand notation for fatty acids: carbon number: double bond number, n – position of the carbon with the first double bond from methyl end; FA – fatty acid, ∑ – the sum, SFA – saturated fatty acids, MFA – monounsaturated fatty acids, PUFAn-6 – polyunsaturated fatty acids of n-6 family, PUFAn-3 – polyunsaturated fatty acids of n-3 family, D9D16 – D9 desaturase for C16 (16:1n-7/16:0), D9D18 – D9 desaturase for C18 (18:1n-9/18:0); + - p<0.05*

Supplementary Table S2

Correlations between fatty acid content and noncholesterol sterols in plasma triacylglycerols

fatty acid	lathosterol/TC (mmol/mmol)	campesterol/TC (mmol/mmol)	b-sitosterol/TC (mmol/mmol)
14:0*	0.0900	0.2396+	0.1997+
16:0	0.0581	-0.0944	-0.0952
16:1n-9	-0.1161	0.1433	0.1254
16:1n-7	-0.1593+	-0.0172	-0.0303
18:0	-0.0724	-0.0335	-0.0114
18:1n-9	-0.0385	-0.0610	-0.0826
18:1n-7	-0.0993	-0.0775	-0.0803
18:2n-6	-0.0266	0.0480	0.0892
18:3n-6	0.0272	-0.0666	-0.0817
18:3n-3	0.2552+	0.1356	0.0661
20:2n-6	-0.1693+	-0.1383	-0.0803
20:3n-6	-0.1316	0.0611	0.1040
20:4n-6	-0.1198	0.1470+	0.1657+
20:5n-3	0.0239	0.1200	0.0887
22:5n-6	-0.1402	0.2648+	0.2327+
22:5n-3	0.0724	0.2578+	0.2140+
22:6n-3	-0.0422	0.1162	0.1224
SSFA	0.0832	-0.0192	-0.0279
SMUFA	-0.0731	-0.0545	-0.0780
SPUFAn-6	-0.0668	0.0661	0.1112
SPUFAn-3	0.1529+	0.2029+	0.1455+
D9D16	-0.2098+	0.0019	-0.0055
D9D18	0.0487	0.0190	-0.0222
20:5n-3/20:4n-6	0.1854+	0.0094	-0.0453
22:6n-3/20:4n-6	0.0325	0.0567	0.0792

*Data for fatty acids are given in molar percentages. Symbols and abbreviations: * -shorthand notation for fatty acids: carbon number: double bond number, n – position of the carbon with the first double bond from methyl end; FA – fatty acid, ∑ – the sum, SFA – saturated fatty acids, MFA – monounsaturated fatty acids, PUFAn-6 – polyunsaturated fatty acids of n-6 family, PUFAn-3 – polyunsaturated fatty acids of n-3 family, D9D16 – D9 desaturase for C16 (16:1n-7/16:0), D9D18 – D9 desaturase for C18 (18:1n-9/18:0); + - p<0.05*

Supplementary Table S3

Correlations between fatty acid content and noncholesterol sterols in plasma cholesteryl esters

fatty acid	lathosterol/TC (mmol/mmol)	campesterol/TC (mmol/mmol)	b-sitosterol/TC (mmol/mmol)
14:0*	0.0864	0.2158+	0.1667+
16:0	0.0976	0.1639	0.1250
16:1n-9	-0.0121	0.1245	0.0797
16:1n-7	-0.0456	0.0391	0.0366
18:0	-0.1245	-0.0081	0.0685
18:1n-9	-0.1193	-0.0178	-0.0178
18:1n-7	-0.0445	0.1602+	0.1475+
18:2n-6	0.0226	-0.1542+	-0.1289
18:3n-6	-0.0262	-0.0654	-0.0410
18:3n-3	0.2198+	0.1860+	0.0962
20:2n-6	-0.0991	0.1043	0.1079
20:3n-6	-0.0545	-0.0663	-0.0274
20:4n-6	0.0055	0.1279	0.1071
20:5n-3	0.0399	0.1403	0.0900
22:5n-6	0.0694	0.1761+	0.1367
22:5n-3	0.0454	0.1914+	0.1437+
22:6n-3	0.0356	0.2196+	0.2026+
SSFA	0.0827	0.1798+	0.1458+
SMUFA	-0.0531	0.0411	0.0242
SPUFAn-6	-0.0015	-0.1389	-0.1089
SPUFAn-3	0.1219	0.2106+	0.1420
D9D16	-0.1255	-0.0420	-0.0198
D9D18	0.0717	-0.0172	-0.0963
20:5n-3/20:4n-6	0.0463	0.1058	0.0464
22:6n-3/20:4n-6	0.0101	0.2137+	0.2117+

*Data for fatty acids are given in molar percentages. Symbols and abbreviations: * -shorthand notation for fatty acids: carbon number: double bond number, n – position of the carbon with the first double bond from methyl end; FA – fatty acid, ∑ – the sum, SFA – saturated fatty acids, MFA – monounsaturated fatty acids, PUFAn-6 – polyunsaturated fatty acids of n-6 family, PUFAn-3 – polyunsaturated fatty acids of n-3 family, D9D16 – D9 desaturase for C16 (16:1n-7/16:0), D9D18 – D9 desaturase for C18 (18:1n-9/18:0); + - p<0.05*

Supplementary Table S4 Selected parameters of liver function

	Group		
**parameter**	**CON** (n = 26)	**HV-HFD** (n = 26)	**reference range**
albumin (g/l)	46.2 ± 2.1[1]	41.0 ± 5.1[a]***	35-53
aspartate aminotransferase (mcat/l)	0.26 ± 0.07	0.40 ± 0.12***	0.10-0.72
alanine aminotransferase (mcat/l)	0.31 ± 0.13	0.45 ± 0.25	0.10-0.78
alkaline phosphatase (mcat/l)	2.06 ± 0.85	1.06 ± 0.31***	0.67/0.58 – 2.15/1.73 M/F
g-glutamyltransferase (mcat/l)	0.61 ± 0.44	0.54 ± 0.30	0.14-0.84 (obesity ↑)

[1] Data are given in average ± SD; [a]ANCOVA adjusted for age and BMI, *** - $p < 0.001$

Supplementary Table S5 Relationships between nutritional parameters and indices of inflammation

**parameter**	albumin (g/l)	TC (mmol/l)	HDL-C (mmol/l)	non HDL-C (mmol/l)	BMI (kg/m^2)
SAA (mg/l)	-0.560[1]**	-0.370	0.049	-0.459*	-0.117
hs-CRP (mg/l)	-0.489*	-0.042	0.242	-0.137	-0.272
oxLDL (mU/l)	0.239	0.722***	0.277	0.639***	-0.043
SAA/apoA-I (mg/g)	-0.539**	-0.359	-0.037	-0.429*	-0.094
HDL-C/apoA-I (mmol/g)	-0.477*	-0.289	0.363	-0.218	-0.228

[1] Spearman rank-order correlation coefficients for dialysis group (n = 26); * - $p < 0.05$, ** - $p < 0.01$, *** - $p < 0.001$
